# Think sink, not source: how vertical farming’s potential is limited by crop breeding

**DOI:** 10.3389/fpls.2025.1621684

**Published:** 2025-10-03

**Authors:** Gertjan Meeuws, Daan Heeling, José M. Mogollón, Jan Willem Erisman, Paul Behrens

**Affiliations:** ^1^ Institute of Environmental Sciences (CML), Leiden University, Leiden, Netherlands; ^2^ Oxford Martin School, University of Oxford, Oxford, United Kingdom

**Keywords:** vertical farming, plant balance model, energy cascade model, sink-limitation, yield estimation, light use efficiency

## Abstract

Vertical farming (VF) could play a role in addressing some global food challenges, yet it requires higher crop yields and lower costs to become viable at large scales. While reductions in capital intensity are required, the need for new cultivars has been largely overlooked. This is partially a result of common crop dynamic models: Energy Cascade Models (ECMs). ECMs derive yield estimates based on assimilate production from incoming energy only, neglecting a plant’s limitations in storing and transporting assimilates. However, VF crops often experience sink-limited as opposed to source-limited conditions. Here, we adapt the ECM into a Plant Balance Model (PBM) that includes sink-limited conditions and show that current VF crop yields for lettuce and tomato are already close to sink-limited conditions. Further improvements in VF lettuce yields from the literature (700 kg m^−2^ yr^−1^) would require an unprecedented 51% decrease in crop cycle time (6.8 days). We estimate potential lettuce and tomato yields at 330 and 369 kg m^−2^ yr^−1^, respectively. However, improving lettuce and tomato yields beyond 230 and 145 kg m^−2^ yr^−1^, respectively, would require temperatures that current genetics do not tolerate. By assessing the sink-limited nature of current VF cultivars using the PBM, we reveal that proactive breeding programs are essential and without them, yields may stagnate very soon and limit future scalability.

## 1 Introduction

Global agriculture faces unprecedented challenges in meeting growing food demands. Agriculture also plays a significant role in driving climate change, biodiversity loss, land use change, and nutrient pollution (Campbell et al., 2017; Tuomisto, 2019; Orsini et al., 2020); with climate change expected to exacerbate global food insecurity (Mirzabaev et al., 2023). To address these challenges, vertical farming (VF) has emerged as one possible solution in some situations. VF provides year-round production of high-quality crops near urban centres, reducing transport emissions and enabling local food supply. They also increase resource efficiency and allow for precise control over environmental conditions which allow for improved crop uniformity, nutritional value, taste, and shelf life. Lastly, they could increase resilience against extreme weather and disruptions in supply chains (Orsini et al., 2020; Van Delden et al., 2021; Despommier, 2024).

VF scalability in part rests on its potential to reduce capital intensity (Appolloni et al., 2022) via technological improvements and resource use efficiency (Benke and Tomkins, 2017; Marchant and Tosunoglu, 2017; Kusuma et al., 2020; Van Delden et al., 2021; Zhu and Marcelis, 2023; Kaiser et al., 2024). To best target these improvements, we require a better understanding of the underlying factors determining crop productivity. The Energy Cascade Model (ECM) (Bugbee, 1992; Volk et al., 1995) is a part of a broader class of dry matter production or radiation-use efficiency models (Marcelis, 1993; de Koning, 1994; Heuvelink, 1996; Higashide and Heuvelink, 2009; Higashide, 2022). These models follow energy moving through the plant to produce biomass (termed the “energy cascade”). The ECM expresses variables such as Light Use Efficiency (LUE) as fractions of their theoretical maximum values, defining the upper limits of potential productivity (Volk et al., 1995).

Cultivar-specific approaches include the source-sink balance (Marcelis et al., 1998) where the source strength (the photosynthesis rate) is defined as the rate of assimilate (carbohydrate) production in plants (Li et al., 2015) and the sink strength is the total potential capacity of a plant to capture and store the assimilates in its organs (Marcelis et al., 1998). In open fields and greenhouses, plants are generally under source-limited conditions, but in vertical farms, photosynthesis can be highly optimised and plants can become sink-limited, unable to store excess assimilates efficiently (Kreuger et al., 2018), ultimately preventing yield gains. These source-sink dynamics have not yet been considered in ECM calculations which limits their application for VF crop production optimisation.

To overcome this, we develop a model for estimating crop yields in VF systems that includes sink-source dynamics called a Plant Balance Model (PBM). We apply the PBM to evaluate potential current and next generation (‘NextGen’) lettuce and high-wire (indeterminate) tomato yields in VF under optimal conditions. We then compare these estimates with existing modeled and experimentally measured yields from literature.

Current yields are estimated using existing cultivars and cropping systems under present-day VF conditions. This includes known constraints related to LUE, Plant Density (PD), and sink capacity. In contrast, NextGen yields reveal the upper limits of productivity illustrating what could be achieved if genetic and technological advancements improve crop performance. This includes enhanced Light Interception (LI), improved LUE, and potential shifts in optimal temperature ranges to accelerate growth without inducing stress (see **Supplementary Table S4** for all input variables). We can then quantify the extent to which NextGen efforts could improve VF yields.

To validate the PBM, we used experimental data from the literature (Karpe et al., 2024), which examined dwarf tomato yields across various planting densities (see **Supplementary Table S2**). This analysis highlights the PBM’s ability to illustrate source-to-sink and sink-to-source dynamics. Theoretically, measured yields should always align with the calculated source- and sink-limited yields. The PBM successfully reproduces measured yields, demonstrating its consistency with observed data and its capacity to represent yield dynamics (details provided in **Supplementary Methods** and **Supplementary Table S2**).

## 2 Materials and methods

**Figure 1:**
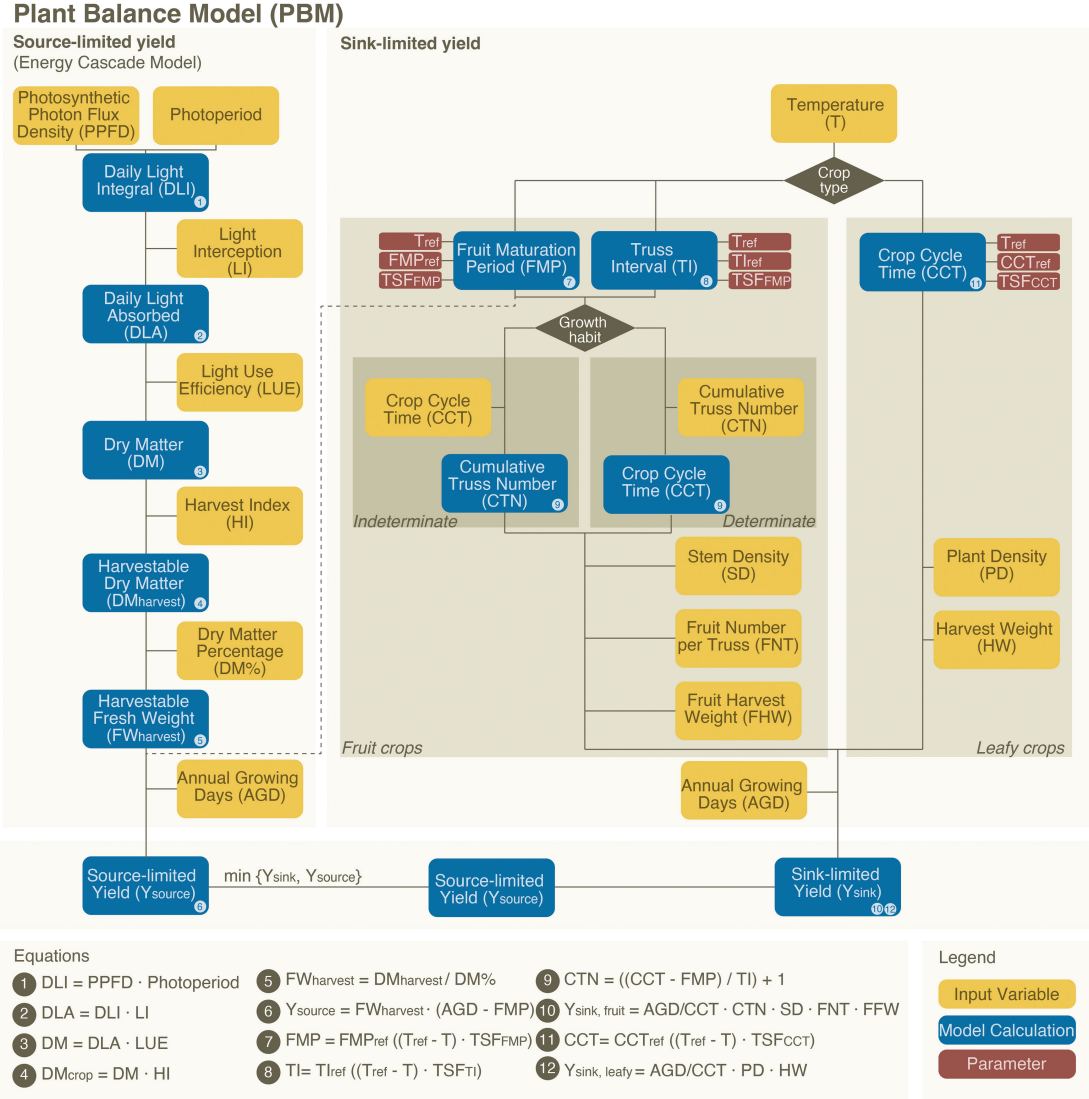
Flowchart of the plant balance model, consisting of the source-limited yield calculation (left) and the additional sink-limited yield calculation (right). For a full overview of the input variables, parameters and model calculations see **Supplementary Table 1**.

The overall PBM framework is shown in **Figure 1**. The PBM builds upon the ECM by integrating both sink-to-source and source-to-sink dynamics. The sink-to-source component of the model, based on the ECM, employs a series of equations (Bugbee, 1992; Amitrano et al., 2020) parameterised by observations. We extend the ECM by calculating the yield based on a plant’s maximum sink capacity. This is primarily determined by the sink strength, the ability of its individual organs to capture and store assimilates from photosynthesis (Marcelis, 1996). The actual yield will be the lowest value of either the source-driven yield (as estimated by the ECM) or the potential sink-limited yield.

We calculate the sink-limited yield for leafy crops by dividing the Annual Growing Days for the farm (AGD) by the Crop Cycle Time (CCT) (the days for a full crop cycle from transplanting a young plant to harvest). This value is then multiplied by the plant density (PD) and the harvest weight of a single plant (HW) (Equation 12 in **Figure 1**). CCT is temperature-dependent and can be estimated using reference-based parameters: the reference temperature (T_ref_), the CCT at the reference temperature (CCT_ref_), and the Temperature Sensitivity Factor for the CCT (TSF_CCT_) (**Figure 1**). To estimate the effect of temperature variation on CCT in lettuce (see Equation 11 in **Figure 1**), we use experimental reference values (Carotti et al., 2021).

For fruit crops, sink-limited yield (Y_sink, fruit_) is calculated from the cumulative truss number (CTN) with the number of stems per m^2^ (SD), the Fruit Number per Truss (FNT) and the Fruit Harvest Weight (FHW), while adjusting for annual yield by dividing the AGD by the CCT (see equations 9 and 10 in **Figure 1**). For these fruit crops, indeterminate crops (e.g., high wire tomato) grow in sequences and can—after being transplanted as a young plant—theoretically grow indefinitely. In a greenhouse setup, the grower determines when to remove the plant—either due to excessive size or at the end of the season—resulting in a variable number of trusses on indeterminate crops. A determinate crop (e.g., some varieties of dwarf tomato) has a fixed growth pattern and produces a predetermined number of trusses. The PBM differentiates between the two (see **Supplementary Figure S1**; **Supplementary Methods**).

From a sink-limited perspective, sink-strength is ultimately dependent on the plant’s growth rate, which increases linearly with temperature (Bensink, 1971; Yan and Hunt, 1999). However, this effect is constrained by the optimal temperature ranges for different crops, beyond which physiological disorders occur [e.g., tip burn in lettuce and disrupted fruit production in tomato (Rudich et al., 1977; Frantz et al., 2004)]. To increase growing speed, crops must be bred with either higher temperature tolerances or higher growing speeds at today’s optimal temperatures. A faster growth rate shortens the CCT in leafy crops and reduces the FMP and TI in fruit crops.

In this study, we evaluate two scenarios— “Current” and “NextGen”. In “NextGen”, to elevate sink-limited yield, we assume cultivars with faster intrinsic growth that sustain performance at 28 °C. Lettuce plant density is increased to 105 plants per m² (Frantz et al., 2004); for tomato, stem density and fruit number per truss rise to 6 stems per m² and 12 fruits per truss. To match the expanded sink capacity, source supply is made non-limiting by increasing PPFD and photoperiod as required and maximising LI via dynamic spacing during the growth cycle. Full inputs are provided in **Supplementary Table S4**.

## 3 Results

**Figure 2:**
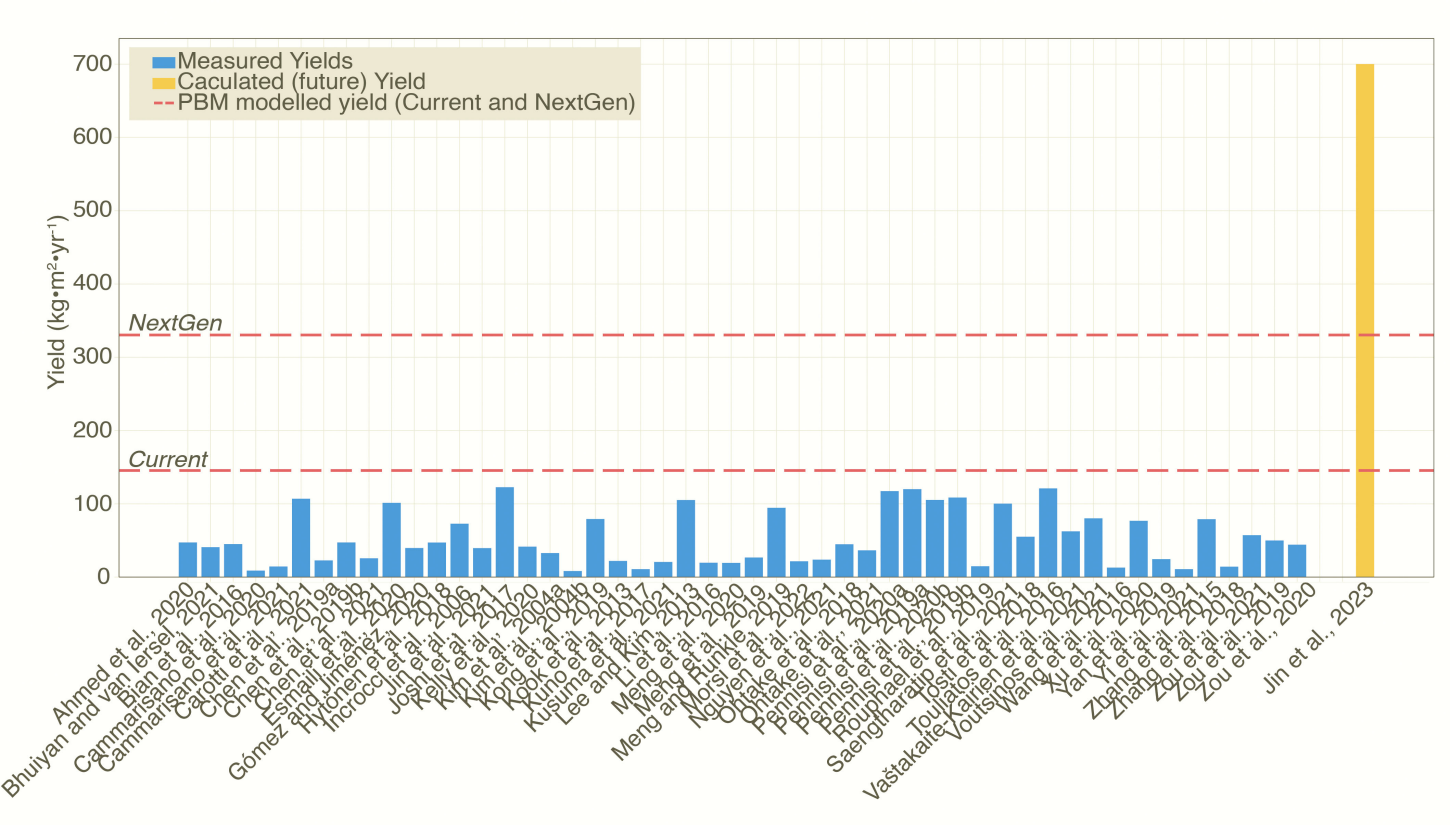
Measured (blue bars) and modelled (yellow bar) lettuce yields in VF setups. (see **Supplementary Table S3** for full details). Red, horizontal dashed lines show the current and NextGen yields.

VF lettuce yields are currently estimated at 146 kg m^−2^ yr^−1^ (Source-limited: 146, Sink-limited: 167). NextGen yields see this climb to 330 kg m^−2^ yr^−1^ (Source-limited: 1049, Sink-limited: 330). While the potential current yield of lettuce is still limited by source-inputs, NextGen yields are projected to be limited by sink capacity. Photosynthetic Photon Flux Density (PPFD) is no longer a limiting factor for NextGen yields, and the sink-limited yield is reached with a PPFD of only 221 µmol m^−2^ s^−1^. These source-sink dynamics explain the differences between the high yields modelled in the literature [at 700 kg m^−2^ yr^−1^ (Jin et al., 2023)], which is more than twice as high as those we find for NextGen yields using the PBM (330 kg m^−2^ yr^−1^) and experimental results (**Figure 2**). We find that the highest measured yields in experiments are close to the potential current yields estimated in the PBM suggesting limited further improvements with current facilities.

We find a current tomato maximum yield of 65.1 kg m^−2^ yr^−1^ (Source-limited: 65.1, Sink-limited: 80.4), with NextGen yields reaching 369 kg m^−2^ yr^−1^. The highest tomato yield for indoor farming experiments achieved in the literature is 47.0 kg m^−2^ yr^−1^ (PPFD ranged from 549 to 893; LUE = 0.5, Dry Matter Percentage (DM%) = 7.5; Annual Growing Days (AGD) = 360) (Wheeler et al., 2008; Eichelsbacher et al., 2025). This is 72% of the PBM’s current estimate, showing that a significant potential for improvements is already possible under current conditions. The current tomato yield is also within the range of estimated yields in high-tech greenhouses (60.2 to 71.6 kg m^−2^ yr^−1^) (Maureira et al., 2022). However, the LUE in VF could be enhanced to near its theoretical maximum (Jin et al., 2023), increasing yields compared to greenhouses. As with lettuce, under current optimal conditions, tomato is still source-limited, but approaches sink-limited conditions for NextGen yields. The required PPFD for NextGen tomato yields under sink limitation becomes 957 µmol m^−2^ s^−1^.

**Figure 3:**
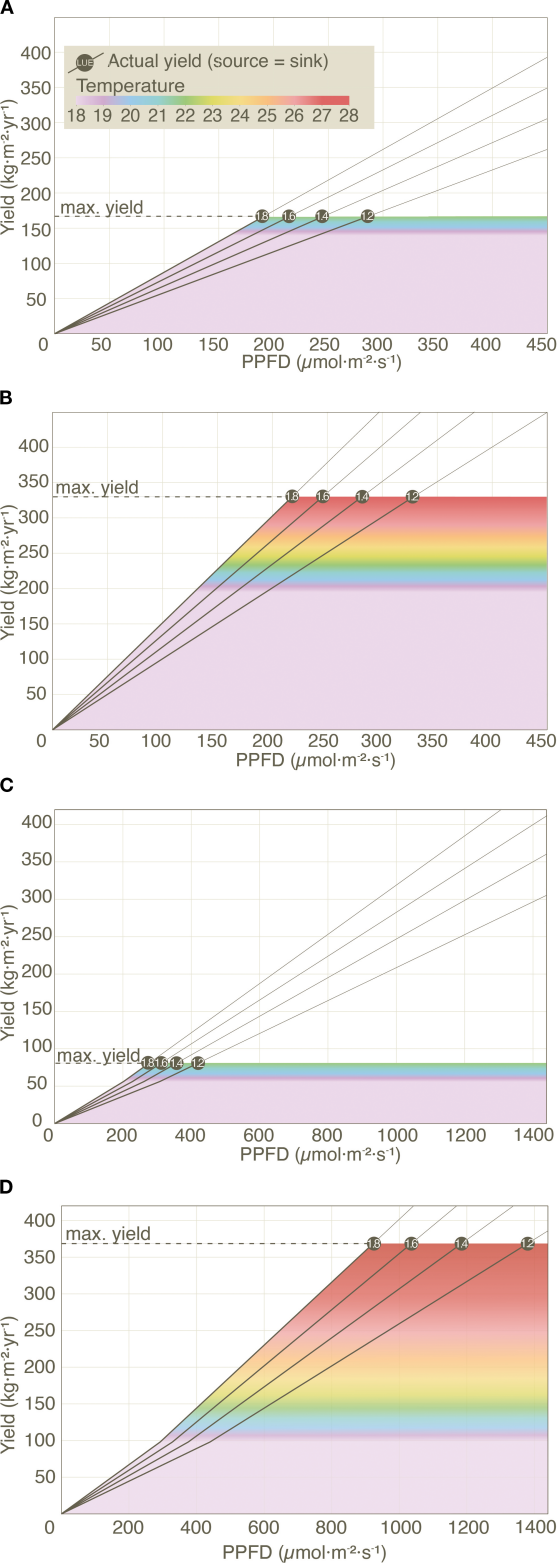
Effect of PPFD, LUE and temperature on (actual) yield in lettuce and tomato under current **(A, C)** and NextGen **(B, D)** scenarios. Diagonal lines represent actual yields across varying temperature (18 to 28 °C) and LUE levels (1.2, 1.4, 1.6, and 1.8 g mol^−1^), with all other input variables held constant (see **Supplementary Table S2**). Unlike lettuce, tomato yields are also influenced by temperature-dependent changes in the Fruit Maturation Period (FMP), causing a distinct shift in the yield trajectory when temperature exceeds 18 °C.

The PBM also highlights several other new dynamics that are not observed in ECMs. For example, lettuce and tomato yields (grown at 22 °C, under current conditions) do not see increased yields as PPFD increases beyond 287 µmol m^−2^ s^−1^ and 432 µmol m^−2^ s^−1^ respectively and this boundary appears even sooner at higher LUE levels (see **Figure 3**).

A second highlight is that yield improvements of 40% in lettuce (from 167 to 234 kg m^−2^ yr^−1^) and 80% in tomato (from 80.4 to 145 kg m^−2^ yr^−1^) are achievable by optimizing PD for lettuce and improving Stem density (SD) and Fruit Number per Truss (FNT) for tomato even without increasing temperature beyond 22 °C (see **Figure 3**). There is a high potential for yield enhancements through these factors alone, without raising temperatures.

Finally, further yield improvements beyond 234 kg m^−2^ yr^−1^ of lettuce and 145 kg m^−2^ yr^−1^ of tomato, requires an increase of growing speed through an increase in temperature (**Figures 3B, D**). Notably, genetic improvements unlock significantly higher yield potentials compared to current capabilities, while also requiring reduced energy input at higher LUEs. This suggests breeding programs would need to focus on improving sink capacity and LUE.

## 4 Discussion

We find significant limitations in achieving the high VF productivities estimated in the literature (Jin et al., 2023). At a market-preferred fresh weight of approximately 125 g per head of lettuce, achieving the highest yield from the literature (700 kg m^−2^ yr^-1^) would demand a drastic increase in both growth rate and plant density. While average Plant Density (PD) from transplant to harvest (i.e., the full growth cycle) could potentially be increased, it approaches a practical limit at about 105 crops m^−2^ on average over the crop cycle (Frantz et al., 2004). As a result, the only remaining variable that can be significantly altered is the Crop Cycle Time (CCT), requiring a transition from trays to harvest of just 6.8 days compared to a 14-day cycle [where LUE of 1.63 g mol^−1^ was used (Pennisi et al., 2019)]. Achieving such a dramatic 51% reduction in Crop Cycle Time (CCT) would require the development of new lettuce cultivars.

These cultivars must either exhibit a substantial increase in growth rate at their current optimal temperature or tolerate higher optimal temperatures that facilitate faster growth. Based on current growth rate-temperature relationships (see Methods), we estimate that sustaining a yield of 700 kg m^−2^ yr^-1^ would require an optimal temperature of approximately 35.7 °C, well beyond what is achievable with present varieties.

Nevertheless, NextGen yield potential is substantially higher than current levels and could provide VF competitivity with at least greenhouse production. Achieving these higher yields will require several large advances. Specifically, research and development efforts will have to shift from targeting modest gains of 20-30% to pursuing yield increases on the order of three to four times the current levels. System-level advancements and breakthroughs in genetic development, reflected in the NextGen scenario, could nearly double lettuce yield and increase tomato yields over 4.5 times compared to current sink-limited constraints.

In ECMs, yield constraints arise from the diminishing relationship between Daily Light Integral (derived from PPFD and Photoperiod) and LUE (Bugbee and Monje, 1992) due to Calvin cycle limitations (Huber et al., 2021). While light saturation thresholds vary (Fu et al., 2012; Huber et al., 2021), our findings indicate that current and NextGen VF systems rarely approach these limits. For lettuce, the highest required PPFD remains well below the lowest reported saturation threshold, and for tomato, only extreme PPFD levels in the NextGen scenario exceed it (**Figure 3D**). This suggests that LUE declines are unlikely to be a practical limitation. Instead, the PBM highlights that sink limitations, rather than light saturation, are the primary constraint of VF crop yields. The PBM also reveals an important nuance often overlooked in literature and industry: the widely assumed 1% increase in light availability leading to a 0.5-1% increase in yield (Heuvelink et al., 2025) only applies under source-limited conditions. Once sink-limitation is reached in the PBM, further increases in light no longer enhance yield.

Some claim VF is approaching theoretical LUE limits (Jin et al., 2023; Eichelsbacher et al., 2025), but this overlooks key factors. The theoretical maximum represents carbohydrate fixation, whereas experimentally measured LUE includes total plant dry weight, which contains 10-20% mineral content (Brouwer, 1962). Adjusting for this discrepancy raises the theoretical LUE to a maximum of 2.17 g mol^−1^, suggesting greater potential for improvement. Additional nuances, including the influence of Harvest Index, can be found in the **Supplementary Discussion**.

We identify sink-limited yields but do not model the physiological adjustments when source-limited yield exceeds sink-limited yield. For example, we do not account for the internal regulatory mechanisms that restore balance within the plant when sink capacity is insufficient. Under sink-limited conditions, plants may reduce photosynthetic efficiency (Plaut and Mayoral, 1984; Li et al., 2015), limit leaf growth (Marcelis, 1991), or redirect excess energy to non-harvestable organs like roots (Arp, 1991). These self-regulating mechanisms and source-sink interactions are not included into the PBM, but can be explored in future work. Additionally, the PBM includes simplifications, such as omitting the light compensation point in source-limited yield estimates and relies on older studies for temperature-growth relationships (see **Supplementary Discussion**). Finally, lack experimental validation for the PBM’s future yield estimates under sink-limited conditions. Current experimental setups rarely achieve strong sink-limited conditions but as technology progresses, we are likely to reach these limits.

Increasing VF sink capacity can be achieved by increasing plant or stem densities, enhancing fruit set in tomatoes by changing inflorescence architecture (Park et al., 2012), or accelerating growth rates without compromising quality. However, unlocking these improvements requires more than just optimizing cultivation techniques, it demands advancements in crop genetics. A dual-track approach is essential: system-level advancements, including better environmental controls and automation, must be complemented by breakthroughs in genetic development in order to further optimize yield and LUE. Only by aligning these two domains can VF achieve the next generation of yield potential.

## Data Availability

The datasets presented in this study can be found in online repositories. The names of the repository/repositories and accession numbers can be found in the article/**Supplementary Material**.
