# Supplementary figures and images for: Think sink, not source: how vertical farming’s potential is limited by crop breeding
Source: Front Plant Sci. 2025 Oct 3;16:1621684. doi: 10.3389/fpls.2025.1621684 (PMC12533277; doi:10.3389/fpls.2025.1621684)

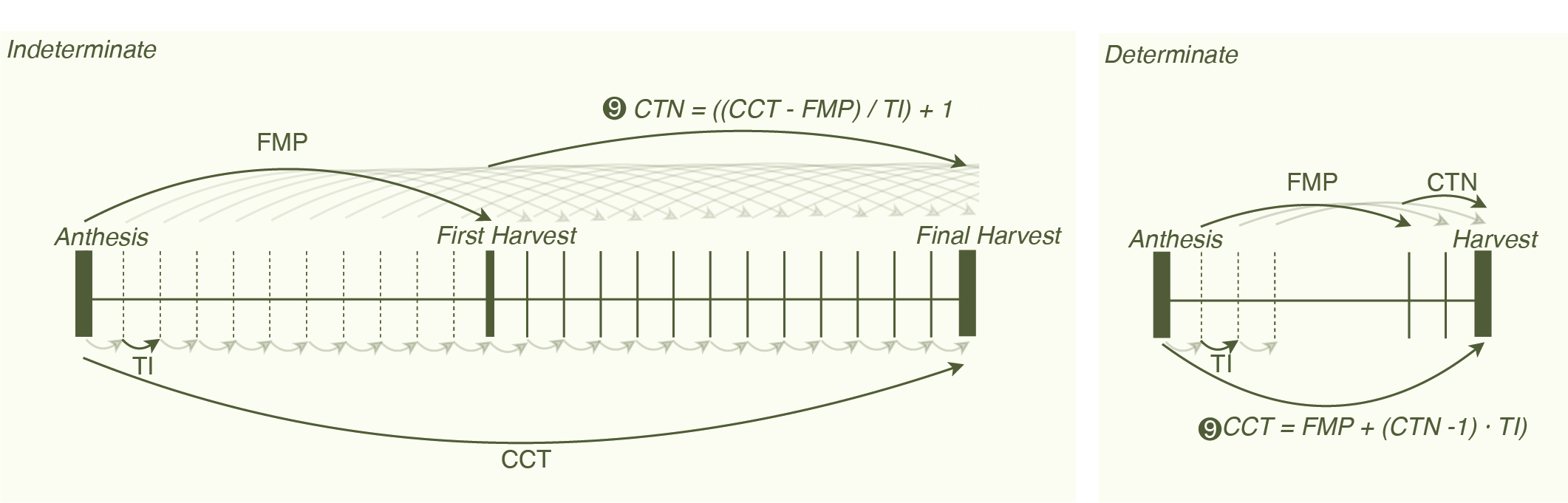

Supplement: Supplementary file 1 [file Image1.jpeg]
