# Supplementary material for: Think sink, not source: how vertical farming’s potential is limited by crop breeding
Source: Front Plant Sci. 2025 Oct 3;16:1621684. doi: 10.3389/fpls.2025.1621684 (PMC12533277; doi:10.3389/fpls.2025.1621684)
Supplement: Supplementary file 2 [file SupplementaryFile1.docx]

Supplementary Material

# Supplementary Information

## Supplementary Methods

# PBM: Determinate and Indeterminate fruiting crops

A tomato crop is transplanted when the first fruit of the plant has reached anthesis. From this point it takes time for the whole fruit to develop and mature until it is harvested, the FMP (in the reference example FMP = 55 days (Koning, 1994). Starting from the first anthesis, the tomato plant grows in units (1 truss + leaves + internodes), each unit takes TI duration to form (in reference example TI = 6.9 days). In turn, each new unit is harvested after the FMP (see Figure 3a and 3b).

In indeterminate crops, the CCT is known by the grower, as the grower decides after how many days the crop is removed. However, the number of trusses grown in that period, the CTN, is unknown. The CTN is calculated by subtracting the FMP from the CCT and dividing it by the TI. Because we already harvest a truss at first harvest, we need to add it to form (CCT-FMP)/TI +1.

In contrast to indeterminate crops, the number of trusses produced during each growing cycle is genetically limited for determinate crops. In this case, CTN is already known. However, CCT remains unknown and can be calculate by FMP, CTN and TI.

These dynamics also have an influence on the source-limited yield. During the adaptation of the ECM into the PBM, we identified that in indeterminate fruit crops not all assimilated energy contributes to harvestable biomass. Unlike determinate fruiting crops or leafy greens, where uniform light intensities can be applied throughout the cycle, high-wire crops undergo distinct phases of light accumulation and reduction.

At the start of the cultivation cycle, plants are introduced with their first flowering trusses already formed, receiving a lower initial PPFD (e.g., 100 μmol/m²·s). Light intensity is then gradually increased as additional trusses develop during the FMP, reaching full production levels (e.g., 400 μmol/m²·s). Conversely, at the end of the cultivation cycle, once the shoot tip is removed, trusses are harvested sequentially, causing a gradual decrease in productive light use. The duration of this light accumulation and reduction period is equal to the 2 x the FMP.

Since, on average, only half of the full PPFD is utilized during these transitional phases, the net productive duration is effectively reduced by 2 x 1/2 × FMP from the AGD. This correction ensures that the PBM accounts only for the days when the crop is in full production, aligning source capacity calculations with realistic light-use patterns. This adjustment is not required for determinate crops (e.g., dwarf tomatoes) or leafy greens (e.g., lettuce), as these crops can receive full PPFD from the start of production without a buildup or reduction phase.

**Parameters (reference values)**

**Lettuce**. CCT_ref_ (22.5 days) and T_ref_ (20 °C), representing the duration from transplanting to reaching the target harvest weight (125g). A PPFD of 200 µmol m^−2^s^−1^, was used as because experiments at higher PPFD were affected by tip burn (Carotti *et al.*, 2021). TSF_CCT_ is set at 1.0 days·°C^-1^ based on the relative difference in number of days after transplanting before reaching the harvest weight of 125g at 20 °C (22.5 days) and 24 °C (18.4 days) (Carotti *et al.*, 2021).

**Tomato**. De Koning (1994) reports a FMP_ref_ of 55 days at a T_ref_ of 21°C. We base the TSF_FMP_ on the difference between the FMP values observed at 17 °C (67.7 days) and 19 °C (57.0 days), resulting in a TSF_FMP_ of 5.4 days·°C^-1^. The TI_ref_ is calculated from the inverse of the number of trusses per day^17^, resulting in a TI_ref_ of 6.9 days at a T_ref_ of 21 °C. The TSF_TI_ is based on the difference in the inversed flowering rate at 17 °C (8.4 days) and 19 °C (7.3 days), resulting in a TSF_TI_ of 0.6.

**Model Validation**

We validated the PBM using experimental data from Karpe, Marcelis and Heuvelink (2024), specifically the ‘75% ground coverage’ and ‘low-density’ experiments. Data points from the study’s supplementary information were used as input variables in the PBM, and the resulting yield estimates were compared with the reported experimental yields (see SI Table 2).

It is important to note that some input variables—such as FTN, FHW, LI, LUE, and HI—were calculated after the experiment and thus depend on the observed yield. As a result, this validation primarily assesses the internal model dynamics rather than its predictive accuracy. Future validation efforts should focus on independent yield predictions using fully empirical input data.

## Software

The development and implementation of the PBM were performed using Python. In addition, WebPlotDigitizer was employed for data extraction from figures, charts, and plots when values were not explicitly reported in the literature. The model is open-source and available at: https://github.com/daanheeling/PlantBalanceModel.

## Supplementary Discussion

**Light Use Efficiency**

**Mineral Content.** The theoretical maximum represents carbohydrate fixation through photosynthesis, whereas experimentally measured LUE reflects total plant dry weight of the plant at harvest per mol incident light. This distinction matters as plant dry weight includes 10-20% mineral content (Brouwer, 1962), which is not accounted for in the theoretical estimate. The theoretical maximum must be critically evaluated, especially since meta-analysis studies (Eichelsbacher *et al.*, 2025) have excluded data points exceeding this threshold. To ensure a valid comparison, either the theoretical maximum LUE should include mineral content (raising it to 1.99–2.17 g mol⁻¹) or experimental LUE should be adjusted to reflect only the organic fraction (lowering the highest recorded LUE from 1.63 g mol⁻¹ to 1.30-1.47 g mol⁻¹). When adjusting the LUE to 2.17 g·mol⁻¹ to include mineral content, the PPFD levels required to achieve the highest projected yields under NextGen scenarios would be 183 µmol·m⁻²·s⁻¹ for lettuce (yielding 330 kg·m⁻²·year⁻¹) and 794 µmol·m⁻²·s⁻¹ for tomato (yielding 369 kg·m⁻²·year⁻¹).

**Harvest Index.** Additionally, Jin *et al.*, (2023) incorporate the harvest index (HI) into their LUE calculations. While this accounts for the proportion of biomass allocated to harvestable organs, it can confuse differences in HI with differences in photosynthetic efficiency. In the PBM, these should be treated separately. A lower HI does not imply lower LUE but instead reflects biomass partitioning between harvestable and non-harvestable organs. The theoretical LUE estimate of 1.81 g·mol⁻¹ assumes an HI of 1, meaning all biomass is harvestable. However, more realistic estimates indicate that lettuce allocates ~5% of its biomass to roots, reducing HI to 0.95 (Jin *et al.*, 2023). Since experimentally observed LUE excludes root biomass, directly comparing it to a theoretical maximum that includes roots introduces inconsistencies. Using our LUE definition based on total DW, the recorded 1.63 g·mol⁻¹ would effectively increase by ~5%, making comparisons more accurate.

**Model Assumption and Limitations**

The PBM’s source-limited yield does not account for the light compensation point, a simplification made for modelling efficiency. As such, the relationship between PPFD and yield passes through the origin, implying that even minimal PPFD levels result in a nonzero yield, which is unrealistic. The light compensation point—the PPFD level where photosynthetic carbon assimilation equals respiratory carbon loss (Nobel and Long, 1985) —sets a threshold below which net photosynthesis is negative, preventing biomass accumulation. The specific PPFD level at which this occurs depends on the LUE. Despite this simplification, we expect minimal impact on our results, as yields in our study are primarily determined under sink-limited conditions, making the source-limited yield threshold less consequential.

Furthermore, some PBM components rely on assumptions and parameters derived from existing literature, particularly older studies. A comprehensive re-evaluation of the parameters used in the PBM to establish the relation between Temperature and plant growth is essential. This effort should focus on incorporating data from modern VF to ensure the model reflects current practices and conditions.

# Supplementary Figures and Tables

## Supplementary Figures

**Figure S1**. Visual representation of the CTN, FMP, TI, and CCT variables in indeterminate fruit crops (left) and determinate fruit crops (right).

## Supplementary Tables

**Table S1.** Definition, units and formulas of the variables in the Plant Balance Model. Input variables are shown in purple, model calculations in blue and parameters in red.

| **Variable** | **Unit** | **Definition** |
| --- | --- | --- |
| PPFD | μmol·m⁻²·s⁻¹ | number of photosynthetically active photons that reach a specific surface every second |
| Photoperiod | hours·day^-1^ | number of hours per day that a plant is exposed to light |
| DLI | mol·m⁻²·day⁻¹ | total photosynthetically active radiation delivered to the growing area in 24 hours |
| LI | % | share of photosynthetically active radiation delivered to the growing area that is intercepted by the plant. |
| DLA | gmol·day⁻¹ | total photosynthetically active radiation that is intercepted by the plants per day |
| LUE | g·mol⁻¹ | efficiency at which intercepted light is converted into dry matter (photosynthesis minus respiration), expressed as the dry weight produced per unit of absorbed light (g·mol⁻¹). |
| DM | g·m^-2^·day⁻¹ | total dry matter produced by a plant per day |
| HI | % | fraction of total dry matter allocated to the harvestable portion of the plant |
| DM_harvest_ | g·m^-2^·day⁻¹ | total dry matter produced allocated to the harvestable portion of the plant per day |
| DM% | % | proportion of the harvestable crop's biomass that is dry matter |
| FW_harvest_ | g·m^-2^·day⁻¹ | total fresh weight produced allocated to the harvestable portion of the plant per day |
| AGD | days·year⁻¹ | number of days per year during which a crop is actively growing |
| GC | days | The total number of days from propagation to harvest per crop. Dependent on Temperature. Changes to model calculation for determinate fruit crops. |
| Temperature | °C | The air Temperature (24h average) during the growth of the crop |
| PD | plants·m^-2^ | The number of plants per square meter |
| HW | kg | The (targeted) Harvest Weight of a single plant. |
| CCT | days | The total number of days from propagation to the end of the harvest period |
| CTN (FMP; TI) | truss·stem^-1^·cycle^-1^ | The number of trusses produced per stem during one growth cycle. Dependent on Fruit Maturation Period (FMP) and Truss Interval (TI). Changes to input variable for determinate fruit crops. |
| FMP | days | The total number of days from anthesis (the point at which the first flower of the truss becomes reproductively mature) to harvest per growth cycle. Dependent on Temperature. |
| TI | days | The total number of Annual Growing Days to form a new growth unit (fruits, internodes and leaves). Dependent on Temperature. |
| SD | stems·m^-2^ | The number of stems per square meter of growing area. If one stem per plant, stem density is equal to Plant Density |
| FNT | fruits·truss^-1^ | The number of fruits per truss. |
| FHW | grams·fruits^-1^ | The targeted Fruit Harvest Weight. |
| T_ref_ | °C | The reference air Temperature (24h average), at which the value of reference parameter (CCT, FMP or TI) is known |
| CCT_ref_ | days | The duration of the Crop Cycle Time at the reference Temperature (leafy crops) |
| TSF_CCT_ | days·°C^-1^ | The effect of Temperature on the Crop Cycle Time (leafy crops) |
| FMP_ref_ | days | The duration of the Fruit Maturation Period at the reference Temperature (fruit crops) |
| TSF_FMP_ | days·°C^-1^ | The effect of Temperature on the Fruit Maturation Period (fruit crops) |
| TI_ref_ | days | The duration of the Truss Interval at the reference Temperature (fruit crops) |
| TSF_TI_ | days·°C^-1^ | The effect of Temperature on the Truss Interval (fruit crops) |

**Table S2**. Model Validation of Sink-limited and Source-limited yield calculation in Dwarf Tomato experiment by Karpe, Marcelis and Heuvelink (2024). A refers to the the 75% ground coverage experiment and B to the low-density experiment.

| **Variable** | **Unit** | **Experiment A** | **Experiment B** |
| --- | --- | --- | --- |
| **Sink** | | | |
| PD | plants·m^-2^ | 41,1 | 18,5 |
| CTN | truss·stem^-1^·cycle^-1^ | 3 | 3 |
| FTN | fruits·truss^-1^ | 8,02 | 7,63 |
| FHW | grams·fruits^-1^ | 7,21 | 7,49 |
| CCT | days | 100 | 100 |
| Temperature | °C | 21 | 21 |
| Yield (sink) per cycle | kg·m^-2^·cycle^-1^ | 7,13 | 3,17 |
| **Annual Yield (sink)** | kg·m^-2^·yr^-1^ | 26,02 | 11,58 |
| **Source** | | | |
| PPFD | μmol·m⁻²·s⁻¹ | 250,0 | 250,0 |
| Photoperiod | hours·day^-1^ | 16 | 16 |
| DLI | mol·m⁻²·day⁻¹ | 14,4 | 14,4 |
| LI | % | 63% | 48% |
| DLA | gmol·day⁻¹ | 9,1 | 6,94 |
| LUE | g·mol⁻¹ | 1,49 | 0,9 |
| DM | g·m^-2^·day⁻¹ | 13,6 | 6,2 |
| HI | % | 53% | 52% |
| DM | g·m^-2^·day⁻¹ | 7,2 | 3,2 |
| DM% | % | 10,2% | 10,2% |
| FW | g·m^-2^·day⁻¹ | 70,7 | 31,7 |
| AGD | days·year⁻¹ | 365 | 365 |
| **Annual Yield (source)** | kg· m^-2^·year⁻¹ | 25,81 | 11,6 |
|  | | | |
| **Annual Yield (sink)** | kg· m^-2^·year⁻¹ | 26,0 | 11,6 |
| **Annual Yield (source)** | kg· m^-2^·year⁻¹ | 25,8 | 11,6 |
| **Annual Yield (measured)** | kg· m^-2^·year⁻¹ | 26,1 | 11,6 |

**Table S3**. Yearly Yield estimation in experiments based on measured data retrieved from Jin *et al.*, (2023).

| **Source** | **DM%** | **Shoot FW** | **Cycle Yield** | **Yield** |
| --- | --- | --- | --- | --- |
|  | = LUE_SDW_ / LUE_SFW_ | = Shoot DW at harvest / DM% | = Shoot FW / 1000 * Density | = 365/plant age * Yield per cycle |
| (Ahmed et al., 2020) | 4,3% | 85,10 | 2,7 | **47,3** |
| (Bhuiyan and van Iersel, 2021) | 3,5% | 48,17 | 4,8 | **40,9** |
| (Bian et al., 2016) | 7,9% | 31,50 | 3,0 | **45,0** |
| (Cammarisano et al., 2020) | 12,5% | 14,40 | 0,7 | **8,9** |
| (Cammarisano et al., 2021) | 5,6% | 23,40 | 1,2 | **14,5** |
| (Carotti et al., 2021) | 2,8% | 363,33 | 9,1 | **106,9** |
| (Chen et al., 2019a) | 4,4% | 100,10 | 2,5 | **22,8** |
| (Chen et al., 2019b) | 5,3% | 43,24 | 1,7 | **47,3** |
| (Chen et al., 2021) | 5,1% | 49,38 | 2,5 | **25,7** |
| (Cope et al., 2014) | 0,0% |  | - | **-** |
| (Esmaili et al., 2020) | 4,1% | 194,67 | 11,1 | **101,3** |
| (Gómez and Jiménez, 2020) | 4,6% | 145,17 | 3,0 | **39,7** |
| (Hytönen et al., 2018) | 4,7% | 100,58 | 4,5 | **47,2** |
| (Incrocci et al., 2006) | 4,3% | 2,30 | 3,0 | **72,8** |
| (Jayalath and van Iersel, 2021) | 4,0% | 62,50 | 0,0 | **0,0** |
| (Jin et al., 2021) | 5,7% | 59,50 | 3,0 | **39,6** |
| (Joshi et al., 2017) | 4,1% | 213,88 | 7,1 | **122,7** |
| (Kelly et al., 2020) | 3,6% | 46,92 | 1,9 | **41,5** |
| (Kim et al., 2004a) | 6,5% | 35,65 | 1,9 | **32,8** |
| (Kim et al., 2004b) | 5,0% | 6,00 | 0,3 | **8,3** |
| (Kong et al., 2019) | 3,4% | 116,00 | 5,0 | **79,2** |
| (Kook et al., 2013) | 4,3% | 4,70 | 1,7 | **22,1** |
| (Kuno et al., 2017) | 5,9% | 22,10 | 0,6 | **10,8** |
| (Kusuma et al., 2021) | 4,0% | 42,50 | 1,2 | **20,7** |
| (Lee and Kim, 2013) | 3,8% | 164,70 | 8,1 | **105,2** |
| (Li et al., 2016) | 3,6% | 42,00 | 1,3 | **19,6** |
| (Meng et al., 2020) | 9,5% | 30,45 | 1,5 | **19,4** |
| (Meng et al., 2019) | 6,9% | 30,45 | 1,5 | **26,7** |
| (Meng and Runkle, 2019) | 5,9% | 1,70 | 2,3 | **94,6** |
| (Morsi et al., 2022) | 6,9% | 127,60 | 3,3 | **21,6** |
| (Nguyen et al., 2021) | 3,7% | 29,43 | 1,8 | **23,8** |
| (Ohtake et al., 2018) | 4,5% | 146,03 | 3,1 | **44,8** |
| (Ohtake et al., 2021) | 6,4% | 84,00 | 2,1 | **36,5** |
| (Pennisi et al., 2020a) | 4,0% | 67,50 | 6,8 | **117,3** |
| (Pennisi et al., 2019a) | 6,1% | 46,03 | 4,6 | **120,0** |
| (Pennisi et al., 2020b) | 5,4% | 60,60 | 6,1 | **105,3** |
| (Pennisi et al., 2019b) | 4,8% | 41,64 | 4,2 | **108,6** |
| (Rouphael et al., 2019) | 4,3% | 48,30 | 0,8 | **14,8** |
| (Saengtharatip et al., 2021) | 4,0% | 316,35 | 10,4 | **100,3** |
| (Spalholz et al., 2020) | 3,2% | 0,00 | 0,0 | **0,0** |
| (Tosti et al., 2018) | 4,1% | 4,92 | 4,7 | **55,1** |
| (Touliatos et al., 2016) | 3,9% | 272,07 | 13,6 | **121,1** |
| (Vaštakaite-Kairien et al., 2021) | 9,4% | 2,13 | 0,9 | **62,5** |
| (Voutsinos et al., 2021) | 4,1% | 212,46 | 6,4 | **80,2** |
| (Wang et al., 2016) | 10,3% | 49,30 | 1,2 | **12,9** |
| (Xu et al., 2020) | 4,6% | 74,23 | 2,5 | **76,8** |
| (Yan et al., 2019) | 4,9% | 34,43 | 1,3 | **24,5** |
| (Yi et al., 2021) | 4,1% | 38,72 | 0,9 | **10,8** |
| (Zhang et al., 2015) | 3,8% | 181,33 | 6,7 | **79,0** |
| (Zhang et al., 2018) | 4,1% | 31,85 | 0,7 | **14,2** |
| (Zou et al., 2021) | 3,8% | 60,26 | 2,4 | **57,2** |
| (Zou et al., 2019) | 6,1% | 52,57 | 2,1 | **49,9** |
| (Zou et al., 2020) | 3,4% | 55,10 | 1,8 | **44,2** |

**Table S4.** PBM overview of input variables for current and NextGen values for lettuce and tomato. *We assume that it takes 5 days/year to accommodate for maintenance and cleaning for tomato crops. ** For both lettuce and tomato, we start from transplanting young plants. For lettuce we also assume dynamically adjusted densities during the growing cycle, to maximize light interception. For tomato in the first phase the LI will not be 100%, however, the Crop Cycle Time (CCT) is set at 360 days. However, we assume an average light interception of 100% over the whole growth phase, even though in the first weeks this will be lower.

| **PBM** | **Input Variable** | **Lettuce** | | **Tomato (indeterminate)** | |
| --- | --- | --- | --- | --- | --- |
|  |  | **Current** | **NextGen** | **Current** | **NextGen** |
| *Source-limited* | PPFD | 250 (Pennisi *et al.*, 2020) | 700 | 350 (Hikosaka *et al.*, 2013; Ke *et al.*, 2023) | 1000 (Hikosaka *et al.*, 2013) |
|  | Photoperiod | 16 (Pennisi *et al.*, 2020) | 20 | 16 (Hikosaka *et al.*, 2013) | 20 (Logendra, Putman and Janes, 1990; Jensen and Veierskov, 1998) |
|  | LI | 85** | 100** | 100** | 100** |
|  | LUE | 1,2 (Jin *et al.*, 2023) | 1,8 (Jin *et al.*, 2023) | 1,2 | 1,8 (Jin *et al.*, 2023) |
|  | HI | 95 | 95 | 65 (Ho, 1984) | 65 (Ho, 1984) |
|  | DM% | 3,5 (Roorda van Eysinga and Smilde, 1971; Heinen, 1994) | 3,0 | 7,5 (Heuvelink, 2005) | 7,5 (Heuvelink, 2005) |
|  | AGD | 365 | 365 | 360 * | 360 * |
| *Sink-limited* | Temperature | 22 | 28 | 22 | 28 |
|  | HW | 125 | 125 |  |  |
|  | FHW |  |  | 40 | 40 |
|  | PD | 75 | 105 (Frantz *et al.*, 2004) |  |  |
|  | SD |  |  | 4 | 6 |
|  | FNT |  |  | 10 | 12 |
|  | AGD | 365 | 365 | 360 * | 360 * |
|  | CCT |  |  | 360 * | 360 * |

# Supplementary References

Bensink, J. (1971) *On morphogenesis of lettuce leaves in relation to light and temperature*.

Brouwer, R. (1962) ‘Distribution of dry matter in the plant’, *Netherlands Journal of Agricultural Science*, 10(No. 5 Special Issue), pp. 361–376.

Carotti, L. *et al.* (2021) ‘Plant Factories Are Heating Up: Hunting for the Best Combination of Light Intensity, Air Temperature and Root-Zone Temperature in Lettuce Production’, *Frontiers in Plant Science*, 11, p. 592171. Available at: https://doi.org/10.3389/fpls.2020.592171.

Eichelsbacher, S. *et al.* (2025) ‘What Is the Limit of Vertical Farming Productivity?’, *Food and Energy Security*, 14(2). Available at: https://doi.org/10.1002/fes3.70061.

Frantz, J.M. *et al.* (2004) ‘Exploring the Limits of Crop Productivity: Beyond the Limits of Tipburn in Lettuce’, *Journal of the American Society for Horticultural Science*, 129(3), pp. 331–338.

Heinen, M. (1994) *Growth and nutrient uptake by lettuce grown on NFT*. 31.50.337291.

Heuvelink, E. (2005) *Tomatoes*. Oxford: CABI. Available at: https://doi.org/10.1079/9780851993966.0000.

Hikosaka, S. *et al.* (2013) ‘Effects of light intensity and amount of supplemental LED lighting on photosynthesis and fruit growth of tomato plants under artificial conditions’, *J. Agric. Meteorol*, 69(2), pp. 93–100.

Ho, L.C. (1984) *Partitioning of assimilates in fruiting tomato plants*, pp. 277–285.

Jensen, E.B. and Veierskov, B. (1998) ‘Interaction between photoperiod, photosynthesis and ethylene formation in tomato plants ( Lycopersicon esculentum cv. Ailsa Craig and ACC‐oxidase antisense pTOM13)’, *Physiologia Plantarum*, 103(3), pp. 363–368. Available at: https://doi.org/10.1034/j.1399-3054.1998.1030309.x.

Jin, W. *et al.* (2023) ‘Light use efficiency of lettuce cultivation in vertical farms compared with greenhouse and field’, *Food and Energy Security*, 12(1), p. e391. Available at: https://doi.org/10.1002/fes3.391.

Karpe, M., Marcelis, L.F.M. and Heuvelink, E. (2024) ‘Dynamic plant spacing in tomato results in high yields while mitigating the reduction in fruit quality associated with high planting densities’, *Frontiers in Plant Science*, 15. Available at: https://doi.org/10.3389/fpls.2024.1386950.

Ke, X. *et al.* (2023) ‘Photosynthetic photon flux density affects fruit biomass radiation-use efficiency of dwarf tomatoes under LED light at the reproductive growth stage’, *Frontiers in Plant Science*, 14, p. 1076423. Available at: https://doi.org/10.3389/fpls.2023.1076423.

Koning, A.N.M. de (1994) *Development and dry matter distribution in glasshouse tomato: a quantitative approach*.

Kreuger, M., Meeuws, L. and Meeuws, G. (2018) ‘Total Indoor Farming Concepts for Large-Scale Production’, in T. Kozai (ed.) *Smart Plant Factory*. Singapore: Springer Singapore, pp. 125–135. Available at: https://doi.org/10.1007/978-981-13-1065-2_8.

Logendra, S., Putman, J.D. and Janes, H.W. (1990) *The Influence of Light Period on Carbon Partitioning, Translocation and Growth in Tomato**. 03044238/90, pp. 75–83.

NOBEL, P.S. and LONG, S.P. (1985) ‘CANOPY STRUCTURE AND LIGHT INTERCEPTION’, *Techniques in Bioproductivity and Photosynthesis*, pp. 41–49. Available at: https://doi.org/10.1016/B978-0-08-031999-5.50014-5.

Pennisi, Giuseppina *et al.* (2020) ‘Optimal light intensity for sustainable water and energy use in indoor cultivation of lettuce and basil under red and blue LEDs’, *Scientia Horticulturae*, 272. Available at: https://doi.org/10.1016/j.scienta.2020.109508.

Pennisi, G. *et al.* (2020) ‘Optimal photoperiod for indoor cultivation of leafy vegetables and herbs’, *European Journal of Horticultural Science*, 85(5), pp. 329–338. Available at: https://doi.org/10.17660/eJHS.2020/85.5.4.

Roorda van Eysinga, J.P.N.L. and Smilde, K.W. (1971) *Nutritional disorders in glasshouse lettuce*. Edited by Institue for Soil Fertility. Wageningen: Centre for Agricultural Publishing and Documentation.

Rudich, J., Zamski, E. and Regev, Y. (1977) ‘GENOTYPIC VARIATION FOR SENSITIVITY TO HIGH TEMPERATURE IN THE TOMATO: POLLINATION AND FRUIT SET’, *BOT. GAZ*, 138(4), pp. 448–452.

Yan, W. and Hunt, L.A. (1999) *An Equation for Modelling the Temperature Response of Plants using only the Cardinal Temperatures*, pp. 607–614.
